# Determinants of premature rupture of membrane in Southern Ethiopia, 2017: case control study design

**DOI:** 10.1186/s13104-018-4035-9

**Published:** 2018-12-27

**Authors:** Yinager Workineh, Shiferaw Birhanu, Sitotaw Kerie, Emiru Ayalew, Manaye Yihune

**Affiliations:** 1 Department of child Health Nursing, College of Medicine and Health Science, Bahir Dar University, Bahir Dar, Ethiopia; 2 Department of Adult Health Nursing, College of Medicine and Health Science, Bahir Dar University, Bahir Dar, Ethiopia; 3 Department of Public Health, College of Medicine and Health Science, Arba Minch University, Arba Minch, Ethiopia

**Keywords:** Premature rupture of membrane, Determinants, Case–control study

## Abstract

**Objective:**

To identify the determinants of term premature rupture of membrane in Southern Ethiopia public hospitals, 2017.

**Results:**

Seventy-five cases and 223 controls women were enrolled for the study. Two hundred eighty-four (95.3%) participants were admitted at the gestational age of above 40, and the rest, 14 (4.7%), were admitted at 37–40 weeks of gestation. The current study identified wealth index and inter-birth interval as preventive predictors, but smoking and hypertension during pregnancy were identified as positive determinants of premature rupture of membrane. This finding is supported by multiple logistic regression analysis result of wealth index (AOR: 0.102, 95% CI [0.033, 0.315]), inter-birth interval (AOR: 0.251, 95% CI [0.129, 0 0.488]), smoking (AOR: 17.053, 95% CI [2.145, 135.6]), and hypertension (AOR: 8.92, 95% CI (1.91, 41.605]). The association between PROM and its determinants indicated that evidence-based interventions should be needed and designed to have very high wealth index, and optimal interbirth interval, and prevent smoking and hypertension during pregnancy to decrease PROM occurrence in the study settings. Hence, we recommended that integration of prevention mechanism of modifiable determinants to the obstetrics health care system will reduce premature ruptures of a membrane.

## Introduction

Premature rupture of membrane (PROM) [1] refers to the disruption of fetal membranes before the beginning of labor which is characterized as painless gush of fluid that leaks out of the vagina (sometimes steady leakage of small amount of watery fluid coming out of the vagina) and a change in color or a decrease in the size of the uterus [2, 3]. PROM which occurs prior to 37 weeks of gestation is preterm PROM, but PROM that occurs after 37 weeks gestation is called term PROM [2].

Approximately 8% to 10% of term pregnancies will experience spontaneous ROM prior to the onset of uterine activity [4]. Ninety-five percent of women with PROM at term will go into labor within 24 h [5], but it is associated with one-third of all preterm births [4]. Moreover, 57% of patients with midtrimester PROM deliver within a week [6]. Babies born preterm can suffer from the complications of prematurity, including death caused by not only due to prematurity but also open membranes provide a path for bacteria to enter the womb [7]. Term and preterm PROM complicates approximately 8% and 2% of pregnancies, respectively. Preterm PROM is associated with 40% of preterm deliveries and 18–20% of perinatal deaths [1, 8–11].

Several studies have shown that the occurrence of PROM is strongly associated with low family income, smoking during pregnancy, coffee drinking, surgery, multiple gestations, poly-hydramnios, gestational hypertension and diabetes mellitus [12–14]. For instance, the study by Gahwagi et al. in Libya highlighted that maternal infection, as well as smoking of the pregnant women and Cocaine intake, were the most frequent cause of PROM [15]. Another study by Hassan and Maryam reported that the most important risk factors for PROM were diabetes and maternal hypertension which were associated with neonatal and maternal complications [16]. The study by Kovavisarach et al. stated that the history of PROM in a previous pregnancy and BMI < 20 is risk factors related to premature rupture of membranes in term pregnant women [17, 18].

Many previous studies on risk factors of PROM were utilized secondary data from health institution which are subjected to miss important variables or information bias. This may lead to un-adjusting of confounders between the independent and dependent variables. Primary or up-to-date data on determinants of PROM is very crucial to give direction for the prevention of PROM in the Ethiopian context. However, the studies that investigated the determinants of PROM by using primary data source are very limited. Then this study will narrow the evidence gap, and it may show findings for responsible bodies of health care system, and to prevent determinants of PROM.

## Main text

### Methods

#### Study setting, design and period

The facility based case–control study design was used in Southern Ethiopia public hospitals from 20th March 2017 to 20th May, 2017. The selected hospitals are the major general and referral hospital which provides services for the mothers and neonates of Gamo Gofa, Yesegen Hizboch, and South Omo zone people.

#### Sample size determination and participant selection

The sample size was determined by using the following assumptions 95% CI, power 80%, non-response rate 10%, case to control ratio, 1:3 and control exposed 21% and OR 0.25 [18]. The final calculated sample size was 301.

Five public hospitals from Southern Ethiopia were selected by simple random sampling method. These Hospitals are a flagship zonal and district hospitals that serve their respective town and other nearby districts and villages. Then based on the number of clients who visited each hospital during the previous 1 year, the total sample size was proportionally allocated to each hospital. Then the number of women who visited each hospital per day was picked in a successive way in delivery wards. Finally, mothers with spontaneous PROM and mothers with rupture of the membrane during labor time were included in the study but mothers with medical or obstetric complications indicating prompt delivery were excluded from the study.

#### Measurements

Five trained midwives collected data from the participants by interviewer-administered questionnaire and physical measurements. The interviewer-administered questionnaire, that contained four parts, namely socio-demographic variables, obstetric history, and maternal problems, was prepared from different articles to address the research objective.

Interpregnancy interval is the interval between the most recent previous childbirth and the starting time of pregnancy for the current child as reported by the mother at the time of contact. It was classified as optimal interpregnancy interval if it is 2–5 years and short if it is below 2 years.

Physical measurements were used to obtain data on MUAC and gestational age of mothers. In this regard, MUAC of each woman was measured at the midpoint between the tips of the shoulder and elbow of the left arm using non-elastic, non-stretchable MUAC tapes. Measurements were recorded to the nearest 0.1 cm. In this study, a poor nutritional status of the mother, defined as MUAC < 23 cm [19]. Similarly, gestational age of participants was confirmed by ultrasonography.

Premature rupture of membrane (PROM), the dependent variable, was confirmed by clinical features (painless gush of fluid that leaks out of the vagina and a change in color or a decrease in the size of the uterus) and sterile speculum examination. Then, all women at any age were categorized as mothers with term PROM (75 cases) and women without PROM (223 controls). Cases are mothers who admitted in labor waiting room and had term premature rupture of membrane before the initiation of labor which is confirmed by clinical features (painless gush of fluid that leaks out of the vagina and a change in color or a decrease in the size of the uterus) and sterile speculum examination. But, controls are mothers who were admitted in delivery ward and had no rupture of membrane before initiation of labor.

#### Data quality assurance

Preparing of a questionnaire in English and translating to Amharic, a pre-test of tool outside the study area, 2 days intensive training of data collectors, continues supervision of data collection process, and carefully checking of collected data on daily basis are the major techniques that were used for keeping of data quality.

#### Data analysis

The collected data were entered, cleaned, coded and analyzed using SPSS version 20. Frequency distribution for selected variables was performed. Cleaning of the data was performed before analysis. To check the statistical significance between the dependent and independent variables, Chi square test was performed. In order to know the crude association between determinants and PROM, crude odds ratio (COR) of PROM with 95% confidence interval [20] was calculated. Those variables, with P < 0.2 from the bivariate analysis were considered for binary logistic regression.

Logistic regression analysis was performed to see the association between predictor and outcome variables. Adjusted odds ratio (AOR) with 95% CI was calculated for each independent variable to check the adjusted association between independent variables and PROM. The statistical significance was set at P < 0.05.

### Results

#### Socio-demographic profile of respondents

During the 2 months period, 75 cases of PROM and 223 non-cases of PROM were enrolled for this study in five hospitals. One hundred twenty (40.3%) respondents were in the age range of 20–24-years. The majority, 96 (32.2%), of the participants attended grade 9–12. One hundred forty-four (48.3%) women were housewives. Ninety-seven (32.6%) of respondents had very rich wealth index. Three (2.7%) participants’ mid upper arm circumference measurement was below 23 cm. Two hundred ninety (97.3%) mothers had singleton gestation (Table 1).

**Table 1:** Socio-demographic profiles of respondents in selected hospitals, 2017 (n = 298)

Variable	Response	Frequency	Percent
Age (years)	15–19	39	13.1
	20–24	120	40.3
	25–29	87	29.2
	30–34	25	8.4
	35+	27	9.0
Religion	Protestant	122	40.9
	Orthodox	144	48.3
	Muslim	21	7.0
	Non-believers	11	3.7
Ethnicity	Gamo	111	37.2
	Gofa	69	23.2
	Wolaita	28	9.4
	Amhara	40	13.4
	Oromo	50	16.8
Educational status	Non-educated	42	14.1
	Read and write	6	2.0
	Grade 1–6	46	15.4
	Grdae 7–8	38	12.8
	Grade 9–12	96	32.2
	Above grade 12	70	23.5
Occupational status	Housewife	144	48.3
	Farmer	29	9.7
	Government employee	57	19.1
	Merchant	68	22.8
Marrietal status	Married	268	89.9
	Not married	15	5.0
	Divorced	10	3.4
	Widowed	2	0.7
	Separated	3	1.0
Residence	Urban	183	61.4
	Rural	115	38.6
Wealth index	Very poor	22	7.4
	Poor	14	4.7
	Rich	70	23.5
	Medium	95	31.9
	Very rich	97	32.6
Mid upper arm circumference	Equal and above 23 cm	295	99.0
	Less than 23 cm	3	1.0
Interpregnancy interval	Optimal	131	44.0
	Short	167	56.0
Gestation	Singleton	290	97.3
	Multiple	8	2.7

#### ANC utilization and obstetrics related problems

All but fifteen women had visited antenatal clinics in hospital, health center, and health post during this pregnancy. Of whom, 108 (36.2%) were visited antenatal clinic four times and above. One hundred fifty (50.3%) participates initiated ANC visit within 4 months of conception (Table 2).

**Table 2:** ANC utilization and risk factors of PROM among respondents in Southren Ethiopia, 2017, (n = 298)

Variables	Response	Frequency	Percent
Utilization of ANC services	No	14	4.7
	Yes	284	95.3
Place of ANC utilization	Hospital	93	31.2
	Health center	172	57.7
	Health post	19	6.4
Number of ANC visit	One visit	17	5.7
	Two visit	86	28.9
	Three visit	73	24.5
	Four and more visit	108	36.2
Time of ANC initiation	16 weeks and below	150	50.3
	After 16 weeks	134	45.3
Provider of ANC	Doctor	12	4.0
	Nurse/midwife	250	83.9
	HEW	22	7.4
Smoking	No	292	98.0
	Yes	6	2.0
Previous surgery	No	289	97.0
	Yes	9	3.0
Infection	No	291	97.7
	Yes	7	2.3
Hydramnios	Normal-hydramnios	286	96.0
	Oligo-hydramnios	4	1.3
	Poly-hydramnios	8	2.7
Gestational age admission	Above 40 weeks	284	95.3
	≤ 40 weeks	14	4.7
Presence of gestational Hypertension	No	286	96.0
	Yes	12	4.0
Types of HDP	Gestational hypertension	4	33.3
	Preeclampsia	5	41.7
	Eclampsia	3	25.0
Presence of gestational DM	No	290	97.3
	Yes	8	2.7

Regarding to obstetrics related problems, 6 (2.0%), 9 (3.0%), 7 (2.3%), 12 (4.0%) and 8 (2.7%) term mothers had a history of smoking, surgery, infection, hypertension and gestational diabetes mellitus, respectively. Two hundred eighty-four (95.3%) mothers developed PROM at gestational age of above 40 weeks. Among mothers who had gestational hypertension the majority, 5 (41.7%), were eclamptic (Table 2).

#### Determinants of term PROM

Multivariable logistic regression indicated that mothers with very rich wealth index were 90% (AOR: 0.102, 95% CI [0.033, 0.315] less likely to experience PROM than mothers who had very poor wealth index. Similarly, participants who had two and above years interbirth interval were 75% (AOR: 0.25, 95% CI: [0.129, 0 0.488]) lower to have PROM than mothers who had below 2 years interbirth interval. On the other hand, smoking and hypertension during pregnancy were the positive predictors of term PROM. The result of this study suggested that mothers who had history of smoking during pregnancy were experienced PROM 17 times (AOR: 17.053, 95% CI [2.145, 135.6]) more likely than participants who did not smoke. Likewise, respondents with hypertension faced PROM 9 times (AOR: 8.92, 95% CI (1.91, 41.605]) higher than women without hypertension (Table 3).

**Table 3:** Determinants of term ROM among term mothers in Southren Ethiopia, 2017 (n = 298)

Variables	Cases	Controls	COR (95% CI)	AOR (95% CI)
Residence				
Urban	43	140	1.797 (0.468, 1.356)	1
Rural	32	83	1.255 (0.737, 2.137)	0.921 (0.399, 2.126)
Interpregnancy interval				
Optimal	26	141	0.309 (0.178, 0.534)**	0.25 (0.129, 0 .488)**
Short	49	82	1	1
Utilization of ANC services				
No	6	8	1	1
Yes	69	215	0.428 (0.143, 1.276)	1.77 (0.50, 7.29)
Smoking				
No	69	223	1	1
Yes	6	0	7.89 (1.49, 41.58)*	17.053 (2.145, 135.6)*
Mid Upper arm circumference				
Equal and above 23 cm	73	222	1	1
Less than 23 cm	2	1	6.08 (0.54, 68.05)	2.887 (0.124, 67.027)
Hydramnios				
Normal hydramnios	66	220	1	
Oligo hydramnios	3	1	6.441 (0.575, 72.135)	9.772 (0.3, 289.1)
Poly hydramnios	5	3	5.368 (1.250, 23.043)	5.723 (0.95, 34.49)
Hypertension during pregnancy				
No	67	219	1	1
Yes	8	4	6.537 (1.909, 22.389)*	8.92 (1.91, 41.605)*
Wealth index				
Very poor	14	8	1	1
Poor	6	8	0.429 (0.109, 1.685)	0.391 (0.085, 1.792)
Rich	5	65	0.268 (0.102, 0.706)*	0.153 (0.044, 0.530)*
Medium	19	76	0.044 (0.012, 0.155)*	0.031 (0.008, 0.123)*
Very rich	31	66	0.143 (0.052, 0.390)*	0.10 (0.033, 0.315)*

* Statistically significant at p < 0.05

** Statistically significant at p < 0.00

### Discussion

Identification of determinants of term premature rupture of membrane by facility-based case–control study in Southern Ethiopia public hospitals was the main objective of this study. The factors of PROM were multifactorial. On the base of this very rich wealth index and 2 and above 2 years interbirth interval were inversely predictor of PROM, but smoking and hypertension during pregnancy found to be positive determinants of it.

In the present study, very rich wealth index was preventive for the occurrence of term premature rupture of membrane. A similar association has to be noted in the past studies [12, 13]. The occurrence of PROM was low in mothers who had very rich wealth index mothers as a result of a reduction of families’ financial stress to obtain balanced diet and health care services. Having such services in turn alleviates the problem of malnutrition, overexertion, poor hygiene, stress, recurrent genitourinary infections, anemia & poor antenatal care. Hence, the risk for occurrence of PROM can be reduced in a very rich group of mothers.

Optimal IPI was also inversely related with PROM. On the reverse, other studies indicated that there was a strong association between short IPI and PPROM [20]. Similarly, short IPI less than 18 months [21] increased the odds of the occurrence of PROM. Reduction of term PROM among women with optimal IPI in the current study can happen as a result of positive effect optimal IPI on Mom’s body. Those participants with optimal IPI could gain enough time to replace nutrient stores before being pregnant again in order to prevent malnutrition during pregnancy which is associated with the occurrence of term PROM. On the other hand, close succession of pregnancies and lactation do not allow the mother sufficient time to restore the nutritional reserves before she is subjected to the stresses of the subsequent pregnancy [22]. Another possible scenario for the association between short IPI and spontaneous preterm labor and PPROM is the persistent inflammatory processes of genital tract (especially endometritis) extending from previous birth to the next pregnancy [23].

Smoking is a significant positive predictor of PROM in the present study. This finding is similar to the other past studies [12, 13, 15, 18, 24, 25]. Smoking leads to decrease of collagen and proteins in membranes by increasing cadmium levels and decreasing the availability of Cu^2+^ for collagen synthesis in amnion mesenchymal cells [26], and also nicotine causes arteriolar constriction leading to uterine decidua ischemia [27] so affecting the integrity of the membranes. This finally leads to premature rupture of membrane.

Similarly, hypertension disorders during pregnancy such as gestational hypertension, preeclampsia, eclampsia, and others were the key determinants of PROM in this study. Women with hypertension had high odds of facing PROM than women without hypertension which is similar with the previous studies [14, 15, 28]. In pre-eclampsia, reactive oxygen species which are generated by oxidative stress, and some pathological conditions that develop during pregnancy and are related to hypoxic stress can affect the elevation of S100B concentration in the amnion [29] and altered production and/or clearance of prolactin from the maternal compartment in the case of hypertensive patients [30] that can bring premature rupture of membrane.

In case of hypertension during pregnancy, there is reduced uteroplacental perfusion as a result of abnormal cytotrophoblast invasion of spiral arterioles and endothelial dysfunction and in turn placental ischemia which brings premature rupture of membrane [31, 32]. This premature rupture of membranes in pregnant women can be happen due to type IV collagen reduction in serum and fetal membrane by Matrix Metalloproteinase-9 [33], and increased cytokine concentrations that may contribute to the endothelial damage [34].

### Conclusion

The association between PROM and its determinants indicated that evidence-based interventions should be needed and designed to have very high wealth index, and optimal interbirth interval, and prevent smoking and hypertension during pregnancy to decrease PROM occurrence in the study settings. Hence, we recommended that integration of prevention mechanism of modifiable determinants to the obstetrics health care system will reduce premature ruptures of a membrane.

## Limitation

This research might be subjected to recall bias since participants might not remember and report past events. The other limitation of this study might be selection biases if there are participants who do not know the initiation of labor.

## Abbreviations

ANC: antenatal care
AOR: adjusted odds ratio
PCA: principal component analysis
COR: crude odds ratio
CI: confidence interval
MUAC: mid upper arm circumference
PROM: premature rupture of membrane
